# Where There’s Smoke, There’s Fire: A Case Report of Turbulent Blood Flow in Lower Extremity Point-of-care Ultrasound in COVID-19

**DOI:** 10.5811/cpcem.2020.10.48809

**Published:** 2020-11-23

**Authors:** Mathew Nelson, Dorothy Shi, Miles Gordon, Yash Chavda, Christina Grimaldi, Tanya Bajaj

**Affiliations:** North Shore University Hospital, Department of Emergency Medicine, Manhasset, New York

**Keywords:** Point-of-care ultrasound, COVID-19, thromboembolism

## Abstract

**Introduction:**

Coronavirus disease 2019 (COVID-19) may predispose patients to increased risk of venous thromboembolism (VTE) due to various pathophysiological mechanisms, including but not limited to endothelial injury, inflammation, cytokine-mediated microvascular damage, and reactive thrombocytosis. A high risk of vessel thrombosis correlates with disease severity, making early identification and treatment of prime consideration.

Although identification of a deep venous thrombosis (DVT) or pulmonary embolism warrants immediate treatment with anticoagulation, trying to predict which COVID-19 patients may be at increased risk for developing these pathologies is challenging.

**Case Reports:**

We present two cases of patients with COVID-19 who had ultrasonographic findings of turbulent blood flow within the deep venous system, without clear evidence of acute proximal DVT, who were subsequently found to have significant VTE.

**Conclusion:**

Point-of-care lower extremity ultrasound has become one of the core applications used by emergency physicians. Typically we perform compression ultrasound for DVT evaluation. This novel finding of turbulent blood flow, or “smoke,” within the deep venous system, may serve as a marker of increased risk of clot development and could be an indication to consider early anticoagulation.

## INTRODUCTION

Coronavirus disease 2019 (COVID-19), the viral illness caused by severe acute respiratory syndrome coronavirus 2 (SARS-CoV-2), has been declared a global pandemic since its initial spread from Wuhan, China. As of June 4, 2020, there have been 6.6 million cases worldwide with more than 389,000 reported deaths.^4^ While many people are asymptomatic or have mild symptoms, patients requiring hospitalization have experienced high mortality rates, often thought to be in part due to undiagnosed venous thromboembolism (VTE). A study of patients hospitalized with COVID-19 showed a cumulative rate of VTE of 21%.^5^ Virchow’s triad of factors contributing to thrombosis consists of hypercoagulability, hemodynamic stasis, and endothelial injury. Patients suffering from COVID-19 are at particularly high risk for developing VTE due to increased platelet activation, endothelial dysfunction, hemostasis, hypoxemic vasoconstriction, and activation of hypoxia-inducible factors.^6^,^7^ [

Although data is limited at this time, several studies have suggested that despite being on anticoagulation, patients have high rates of VTE; either deep venous thrombosis (DVT) or pulmonary embolism (PE) were found in up to 27% of COVID-19 polymerase chain reaction (PCR) positive patients.^7^,^8^ Patients with COVID-19 also have high rates of other prothrombotic complications such as clotting within the continuous renal replacement therapy (CRRT) or extracorporeal membrane oxygenation (ECMO) circuits.^7^ Although patients are suffering from VTE despite being on anticoagulation, a preliminary study from the Mount Sinai health system suggests that systemic anticoagulation may still be associated with improved outcomes.^8^

DVT is a common pathology with an incidence of approximately 100 persons per 100,000 each year in the general population, with higher rates in advancing age.^9^ Early recognition and treatment of VTE is important as it is associated with a 28-day case-fatality rate of 11% for a first-time VTE in those over 45 years old.^10^ Diagnosis of VTE begins with risk stratification based on history and physical exam findings, followed by imaging modalities including lower extremity ultrasonography or computed tomography angiogram (CTA).

Although there are many ultrasonographic findings suggestive of a thrombus, we report an ultrasonographic finding of slow, turbulent blood flow, or “smoke,” in the deep venous system in two patients presenting with signs and symptoms of COVID-19. In the first case, a patient with “smoke” but an otherwise negative point-of-care ultrasound (POCUS), was found to have a large PE; in the second case the patient was appropriately discharged but returned to the emergency department (ED) days later and ultimately died of a suspected massive PE. Similar findings suggestive of slow venous flow have been reported by Jensen et al^11^ in oncology patients. In this paper, the ultrasonographic findings were correlated with a near doubling of the risk of developing a DVT. As there is an increased risk of VTE with COVID-19, the addition of ultrasonographic findings of slow, turbulent flow in the deep veins may suggest that providers may need to start thromboprophylaxis earlier or, at a minimum, ensure increased surveillance and follow-up of these patients.

## CASE REPORTS

### Case 1

**Image 1:**
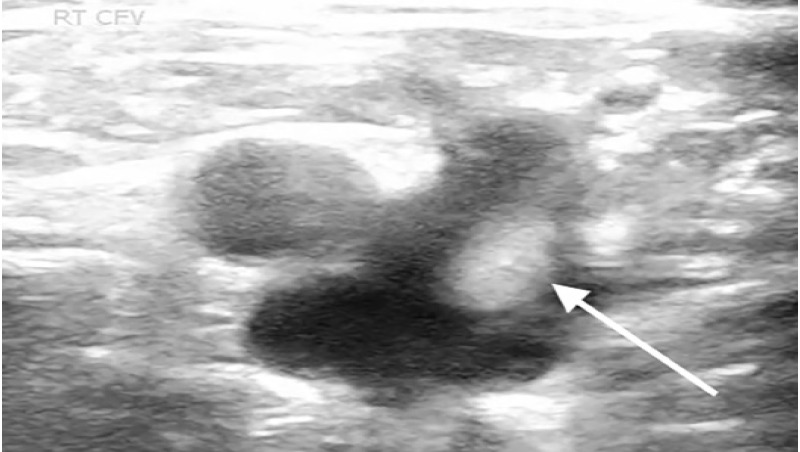
Point-of-care ultrasound of the lower extremity with arrow identifying hyperechoic turbulent flow in the saphenous-common femoral vein junction.

**Image 2:**
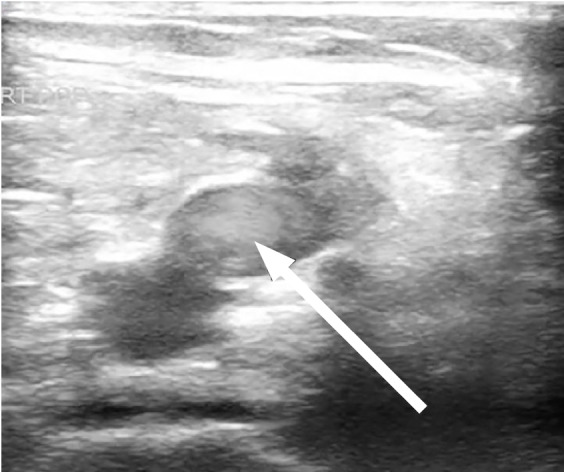
Point-of-care ultrasound of the lower extremity with arrow identifying hyperechoic turbulent flow in the popliteal vein.

A 61-year-old male with no known past medical history, presented with fevers and shortness of breath for three weeks. The patient was seen at another hospital one week prior where he tested positive for COVID-19. On physical exam the patient was ill-appearing, with an increased work of breathing and an oxygen saturation of 77% on room air, which improved to 95% on a 15-liter (L) non-rebreather mask. The rest of his exam was otherwise unremarkable. Laboratory evaluation showed an elevated D-dimer 1556 nanograms per milliliter (ng/mL) [<= 229ng/mL D-dimer units], elevated high-sensitivity troponin of 360 ng/L [reference range 0–51 ng/L], and elevated serum pro-brain natriuretic peptide of 2209 picograms (pg)/mL) [ref range 0–300 pg/mL]. Chest radiograph demonstrated patchy bilateral opacities. Point-of-care lower extremity duplex compression ultrasound demonstrated adequate compression but multiple areas of turbulent flow within the deep venous system (Image 1 and 2).

**CPC-EM Capsule**

What do we already know about this clinical entity?

Little is known about the link between Coronavirus Disease 2019 (COVID-19) and increased turbulent blood flow, or “smoke,” in the deep venous system.

What makes this presentation of disease reportable?

This novel finding of increased turbulent blood flow in two COVID-19 patients suggests that the presence of “smoke” may mark an increased risk for clot development.

What is the major learning point?

Turbulent flow may be indicative of ongoing thromboembolic disease and may be used as a predictor for thromboembolic events in COVID-19 patients.

How might this improve emergency medicine practice?

In patients with COVID-19, bilateral lower extremity duplex demonstrating turbulent flow may lead to earlier detection and treatment of thromboembolic disease.

A CTA of the chest showed a PE within the right main pulmonary artery extending into the right upper lobar and segmental pulmonary arteries. The patient was started on therapeutic heparin. He progressively worsened, becoming hypotensive, tachypneic, and acidotic. He was subsequently intubated several days later and ultimately expired.

### Case 2

**Image 3:**
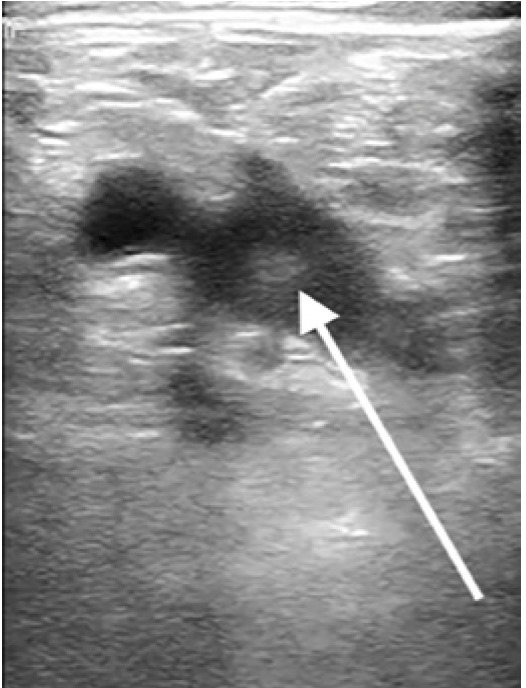
Point-of-care ultrasound of the lower extremity with arrow identifying hyperechoic turbulent flow in the common femoral vein.

A 54-year-old male with a history of diabetes, hypertension, hyperlipidemia, hypothyroidism, coronary artery disease, and a DVT, who had not been on anticoagulation at the time due to gastrointestinal bleeding, presented to the ED with fever, chills, body aches, and chest tightness as well as worsening bilateral calf pain. The patient reported feeling ill for the prior 10 days with progressively worsening symptoms of fatigue, fever, myalgias, and diarrhea. The patient was a hospital employee and tested positive for COVID-19 four days prior to the ED visit. Upon arrival to the ED he was found to be hypoxic to 90% on room air, tachypneic with a respiratory rate of 20 breaths per minutes, tachycardic with a heart rate of 102 beats per minute, afebrile with oral temperature of 98.7 degrees Fahrenheit, and blood pressure of 125/75 millimeters mercury. On physical exam the patient was ill-appearing with increased work of breathing, decreased breath sounds bilaterally, and bilateral calf tenderness to palpation. Laboratory evaluation showed an elevated D-dimer of 465 ng/ml (with a normal cutoff value of 229 ng/ml). A point-of-care lower extremity duplex compression ultrasound demonstrated turbulent blood flow in the right lower extremity (Image 3).

A right calf vein thrombosis was noted distal to the trifurcation. (This was an incidental finding as calf vein imaging is not part of the compression ultrasound protocol.) A CTA was negative for PE but showed bilateral peripheral ground-glass opacities. The patient was placed on supplemental oxygen, started on enoxaparin and admitted to the hospital. He was admitted for four days with improvement and discharged home on anticoagulation. The patient returned to the ED five days later with worsening shortness of breath, hypoxia, and chest pain. A CTA again demonstrated no PE. D-dimer at that time was 31,876 ng/ml. During the second admission, a subsequent comprehensive ultrasound demonstrated a persistent calf vein thrombosis, and the patient’s D-dimer continued to downtrend but was significantly higher than on the prior visit. Radiology did not comment on the turbulent flow finding; however, upon retrospective review of these images, the patient continued to have turbulent flow in the deep venous system of the lower extremity. His oxygen requirements continued to escalate along with the deterioration of his hemodynamics. A repeat bedside echocardiogram showed evidence of right heart strain with an enlarged right ventricle, septal bowing, and low tricuspid annular plane systolic excursion. He was given tissue plasminogen activator for a suspected massive PE, without significant improvement and ultimately expired in the intensive care unit.

## DISCUSSION

Venous thromboembolism diagnosis and treatment is a common challenge among emergency providers. Studies have continually attempted to investigate how lab values, such as fibrinogen and D-dimer, may help risk-stratify patients at risk for VTE and predict mortality rates.^12^ Traditionally in the ED, D-dimer cutoff levels are used to decide whether further imaging such as lower extremity ultrasound or CTA chest is warranted. In COVID-19 patients, D-dimer levels seem to be intrinsically elevated, thereby making it a less useful tool to risk-stratify COVID-19 patients. There have been suggestions to modify the D-dimer cutoff levels in order to adapt to COVID-19 pandemic; however, no conclusion has been established to date.^13^ To our knowledge, this case report is the first to suggest how an ultrasonographic sign can be used as an adjunct to risk-stratify COVID-19 patients for possible VTE events.

Venous stasis as it relates to VTE has been recognized for years but has been studied in only a few subgroups. Jensen et al^11^ described slow venous flow state on ultrasound in oncology patients and how it correlates with higher risk of developing DVTs. We describe this finding in the context of COVID-19 patients. In several COVID-19 patients in our ED, emergency ultrasound fellowship- trained physicians found the ultrasonographic finding of static, turbulent flow, or “smoke,” in the deep venous system without clear evidence of a proximal DVT. In some of these patients we identified this finding in bilateral lower extremities. Typically a DVT is ruled out with a comprehensive duplex ultrasound with Doppler to evaluate for compressibility and normal spectral waveforms^14^ POCUS has been used successfully in evaluating the presence of proximal lower extremity thrombosis with compression ultrasound.^15^

We hypothesize that ultrasonographic “smoke” can be an early indicator of venous stasis in COVID-19 patients, which would be associated with increased risk of developing future VTE. Even though evaluation of “smoke” is not a typical component of point-of-care lower extremity ultrasound, we suggest that emergency providers be aware of this finding and its potential prognostic significance for possible development of VTE. In addition, the finding of “smoke” may suggest that closer surveillance of COVID-19 positive patients for VTE may be necessary.

## CONCLUSION

Multiple studies have highlighted the higher risk of thrombotic events in COVID-19 patients and have attempted to correlate laboratory values with venous thromboembolism risk. We propose the addition of the ultrasonographic finding of slow turbulent flow, or “smoke,” as a risk factor for VTE and should prompt the provider to highly consider thromboprophylaxis, or at the very least lower the threshold for further imaging to evaluate for significant VTE.
